# Acupuncture for the Treatment of Oculomotor Paralysis: A Pilot Randomised Controlled Trial

**DOI:** 10.1155/2016/3961450

**Published:** 2016-05-24

**Authors:** Jia-Qi Bi, Wei Li, Qi Yang, Bao-lin Li, Qing-Gang Meng, Yu-fu Liu

**Affiliations:** ^1^Department of Study Center, The First Hospital of Harbin City, Harbin Medical University, Harbin 150001, China; ^2^Department of Study Center, Medical Science Institute of Harbin, Harbin 150001, China

## Abstract

This study consisted of a single centre randomised controlled trial with two parallel arms: an acupuncture group (*n* = 20) with 27 affected eyes and a sham group (*n* = 20) with 23 affected eyes. Participants in the acupuncture group received acupuncture treatment once daily, three times weekly for four weeks. Participants assigned to the control group received sham acupuncture, the same protocol as that used for the acupuncture group but without insertion of needles into the skin. The primary outcome measure was the cervical range of motion (CROM) score. Secondary outcome measures were the palpebral fissure size, response rate, and adverse events. All 40 participants completed the study. In the comparison of acupuncture and sham acupuncture, a significant difference was observed between acupuncture and sham acupuncture in CROM score (21.37 ± 15.16 and 32.21 ± 19.54, resp.) (*P* < 0.05) and palpebral fissure size (7.19 ± 2.94 and 5.41 ± 2.45, resp.) (*P* < 0.05). Response rate was also significantly different in the acupuncture group (*P* < 0.05). No adverse events were reported in both groups in this study. In summary, it was demonstrated that acupuncture had a feasibility positive effect on oculomotor paralysis.

## 1. Introduction

Ophthalmoplegia is a serious problem among patients with diabetes mellitus, stroke, cerebral tumours, and traumatic brain injury [1–3]. In a survey of 6,765 hospitalised subjects, ophthalmoplegia was identified in 27 patients (0.4%) [4]. Ophthalmoplegia commonly affected the oculomotor nerve and abducens nerve (VI) [5–9]. Of those, the oculomotor nerve was the most frequently affected and it was reported that isolated oculomotor paralysis (OP) accounted for the majority of patients (59.3%) [4]. In addition, these patients may also develop diplopia [10].

A wide range of modalities have been used to treat OP, such as tetracycline [11], Methycobal [12], and vitamin B12 [13, 14]. However, all of them had unsatisfied efficacy and adverse events. Acupuncture therapy is used to treat various conditions, such as pain [15–17] (including neck pain [18, 19], knee pain [20–22]), stroke rehabilitation [23], urinary dysfunction [24, 25], pressure ulcers [26, 27], and OP [28].

Several clinical studies support the idea that acupuncture may help treat the symptoms of OP [12, 29, 30]. To the best of our knowledge, there are no randomised controlled trials and only studies of clinical observations have evaluated the efficacy of acupuncture in treating OP. In addition, the current evidence is poor due to small sample size, poor quality of results, rare follow-ups, and no reporting of adverse events. Considering these methodological flaws, a pilot randomised controlled, four-week clinical trial was conducted to evaluate the possible benefit of acupuncture for OP. It was primarily hypothesised that acupuncture is superior to sham acupuncture in treating patients with OP.

## 2. Patients and Methods

### 2.1. Design

This study was a two-parallel-arm randomised controlled trial. The trial was conducted at clinical research centres in China in accordance with the Declaration of Helsinki and the Guidelines for Good Clinical Practice: the Evidence-Based Medicine Centre of The First Hospital of Harbin City. Inclusion took place between May 2014 and September 2015. The study was approved by the Medical Ethical Committee of The First Hospital of Harbin City. Eligible patients were randomly allocated to the acupuncture group or the sham group at a 1 : 1 allocation ratio and received treatment for four weeks, with one month of follow-up.

### 2.2. Inclusion and Exclusion Criteria

Participants were aged 18–75 years with OP [31, 32]. In addition, no acupuncture was performed within the month prior to entry into the study; and an informed consent document was signed. Exclusion criteria included etiology of aneurysm, infection, inflammation, other major medical illnesses such as multiple sclerosis, myasthenia gravis, temporal arteritis, pseudotumour cerebri, history of childhood strabismus, and degenerative neurologic disorders, rejection of acupuncture treatment, and failure to accept the completion of clinical treatment.

### 2.3. Randomisation and Allocation

Randomisation was conducted using a computerised number generator through the stratified block randomisation method of the SAS package (Version 9.1.3; SAS Institute Inc., Cary, North Carolina, USA) by a statistician with no clinical involvement in the trial. After qualifying, patients were assigned to acupuncture or sham acupuncture therapy by investigators. The allocation was concealed in sequentially numbered, opaque, sealed envelopes containing the randomisation assignments. The participants, outcome assessors, and statistician were blinded throughout the study period. However, the acupuncturists were not blinded due to the nature of the intervention.

### 2.4. Participants and Recruitment

The plan was to conduct the research in The First Hospital of Harbin City. In preparing for this research, it was identified that the centre offered acupuncture or sham acupuncture treatment to 40 people between May 2014 and September 2015. This enabled a fair test of the feasibility criteria and, if recruitment was good, permitted a reliable calculation of the effect size of the treatment for computing a later sample size. Patients who were accepted for both acupuncture and sham acupuncture treatment were informed about the research and given an information sheet. If the patients agreed to participate, consent was taken at the next appointment. After clinical assessment, subjects were randomised to receive either acupuncture or sham acupuncture, which was delivered by the investigators, all of whom were fully trained in its delivery.

### 2.5. Intervention

The comparison groups were acupuncture and sham acupuncture. Sterile and disposable acupuncture needles (0.20 mm × 25 mm) were used. Participants in the acupuncture group received acupuncture at acupoints Chengqi (ST 1); Yuyao (EX HN4); Taiyang (EX-HN5); Shangming; and bilateral Hegu (LI4) for 20 min daily, three times weekly for four weeks. Participants assigned to the control group received sham acupuncture, once daily, three times weekly for four weeks. The same team carried out the sham acupuncture treatment at the same acupoints and according to the same protocol as that used for the acupuncture group but without insertion of needles into the skin. An empty needle tube was taped to the skin at acupoints to produce sensations similar to those of needle insertion, after which the needles were inserted into a piece of adhesive foam taped to the skin.

### 2.6. Clinical Assessments

The primary outcome measure was the cervical range of motion (CROM) score. Secondary outcome measures were the palpebral fissure size, response rate, and adverse events.

#### 2.6.1. Primary Outcome Measure

Diplopia was recorded as either present or absent for each subject and was scored using the CROM method. A higher score reflected a more serious disease state. This method used real-world targets in free space at two testing distances with the scoring weighted towards primary position and reading [33]. The CROM method was proposed as a reliable, simple, and inexpensive alternative to the Goldmann method that permits diplopia to be evaluated at a distance and near fixation [33, 34]. The initial score ranged from 0 (no diplopia) to 25 (constant diplopia) and could easily be rescaled from 0 to 100 by multiplying the score by 4.

#### 2.6.2. Secondary Outcome Measures

Palpebral fissure size (from midpoint of upper eyelid to midpoint of lower eyelid) of affected eyes was measured by simple and noninvasive digital photography and digital image analysis [35]. A higher score reflected better recovery of the affected eyes. The digital photographs were two-dimensional images where the complexity of light was represented as a pixel matrix of fixed and known location values and even colour intensity. Digital photographs were taken at a standardised distance, framing the face centrally, with the gaze lasting a few seconds, using a digital camera (Sony Cybershot DSC P32, *f* = 1/2.8, 1632 × 1224 pixels of resolution, RGB 24 bits, ISO 100). Images were then saved as JPEG files. Digital photographs were taken at a 60 cm distance from the patient, by the same photographer in the same room. The camera was positioned at eye-height, under the same artificial fluorescent lighting. The images were analysed in Image J 1.41v software.

The response rate was assessed by the following criteria [36]. Complete response was as follows: eyeball moving normally, without appearance of squint, double vision, ptosis of the upper eyelid, and headache, and palpebral fissure returning to normal size. Partial response was as follows: eyeball moving normally, however, with occasional suffering from double vision and headache, and improvements shown in the size of the palpebral fissure and in ptosis of the upper eyelid. Nonresponse was as follows: no improvement in the symptoms of ophthalmoplegia.

### 2.7. Sample Size and Analysis

As a feasibility study, it was estimated that a sample of 40 participants with 20 in each group, assuming a drop-out rate of 20%, would be sufficient to provide data to answer the study questions [37, 38]. Data was analysed by a statistician, blinded to the allocation of groups, using SPSS 17.0 statistical software packages. Levels of significance were reported at *P* < 0.05; the Mann-Whitney *U* test was used for the CROM score and palpebral fissure size and the chi-square test for response rate. The data analysis of baseline characteristics, as well as the primary and secondary outcome measures, is based on the intention-to-treat (ITT) principle, which is defined as the participants who are randomised and received at least one treatment session. In addition, analysis of covariance was also conducted for possible baseline incomparability.

## 3. Results

**Figure 1:**
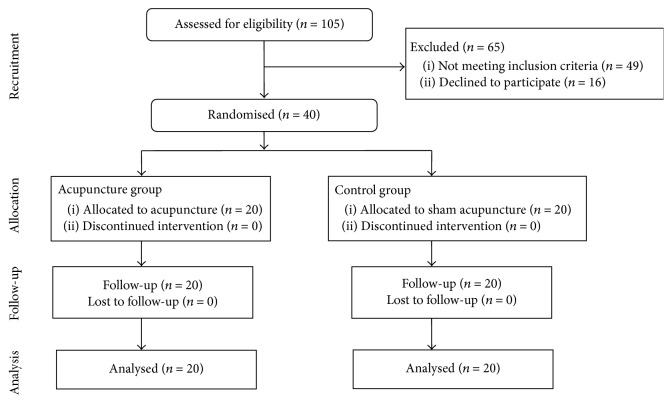
Flow of participants through the trial.

Initially, 105 participants entered the study. Of these, 65 individuals were excluded, as 49 did not meet study criteria and 16 refused to participate. Therefore, 40 participants were randomised and all 40 patients completed the study and were included in the analysis (Figure 1). Characteristics of the study sample are shown in Table 1. The two groups did not differ significantly in sociodemographic and baseline characteristics (Table 1).

**Figure 2:**
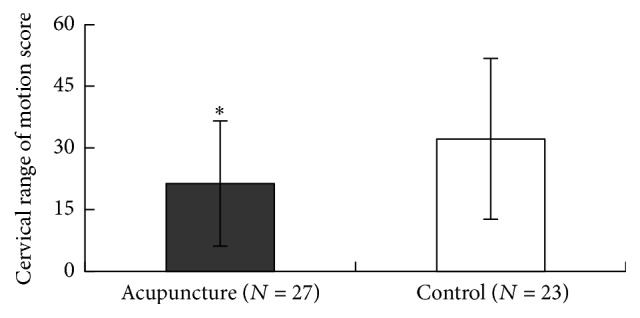
Comparison of cervical range of motion score between two groups. Significant difference was found between the two groups (^*∗*^ *P* < 0.05).

At baseline, the mean CROM score (±SD) was 55.16 (±21.24) in the acupuncture group (27 affected eyes) and 53.28 (±20.03) in the sham acupuncture group (23 affected eyes) (*P* > 0.05). After four weeks of treatment, the mean for the acupuncture group was 21.37 (±15.16) compared to 32.21 (±19.54) for the sham acupuncture group, with statistical difference between the two groups (*P* < 0.05) (Figure 2).

**Figure 3:**
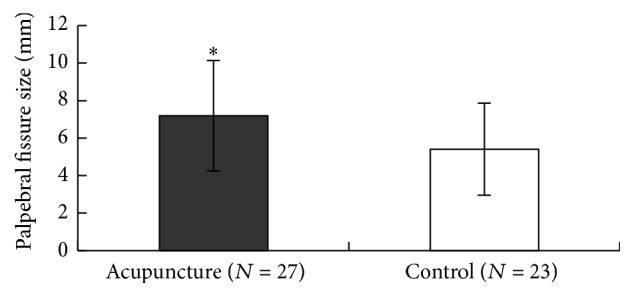
Comparison of palpebral fissure size between two groups. Significant difference was found between the two groups (^*∗*^ *P* < 0.05).

At baseline, the mean palpebral fissure size (±SD) of each of the two groups was also similar: 3.54 (±1.08) for acupuncture (27 affected eyes) and 3.27 (±1.12) for sham acupuncture (23 affected eyes) (*P* > 0.05). After four weeks, the palpebral fissure size was increased to 7.19 (±2.94) for acupuncture and 5.41 (±2.45) for sham acupuncture. Thus, a statistical difference between the palpebral fissure size of the two groups was found after a four-week treatment (*P* < 0.05) (Figure 3).

Response rate at week 4 also showed a statistical difference between the acupuncture group (27 affected eyes) and the sham acupuncture group (23 affected eyes) (*P* < 0.05) (Table 2). In addition, no adverse events were reported in both groups.

## 4. Discussion

Effective salvage regimens for OP are clearly needed. In this study, the use of acupuncture was evaluated, which is not usually employed in first-line regimens. However, acupuncture has demonstrated positive efficacy in OP [12, 28–30, 39, 40].

Several clinical studies reported that acupuncture may be efficacious in OP. Three small trials conducted in China demonstrated overall response rates of 91.6%, 94.3%, and 96.9%, respectively [28–30]. The other clinical observation reported complete response rate of 33.3% and partial response rate of 40.0% [39]. Another publication found that acupuncture showed positive efficacy in OP with overall response rates of 95% when compared to 70% in the control group [40].

Currently, there have been no randomised controlled trials of acupuncture for patients with OP, and, as a result, this study was designed and conducted. This study aimed to conduct a pilot clinical trial of acupuncture for the treatment of OP and to examine its potential effect in future episodes. In addition, it also provides the possibility of a larger clinical trial. The data sheds new light on acupuncture, especially for acupuncture therapy in OP.

The results of this study have demonstrated that acupuncture is efficacious in OP patients with no adverse events.

## Figures and Tables

**Table 1:** Baseline characteristics of participants at trial entry.

	Variable	Group		*P *value
		Acupuncture (*n* = 20)	Control (*n* = 20)	
Mean age (SD) (year)		58.09 (17.24)	61.51 (15.38)	0.51

Sex	Male	9 (45.00%)	12 (60.00%)	0.34
	Female	11 (55.00%)	8 (40.00%)	0.34

Employment	Employed	2 (10.00%)	3 (15.00%)	0.63
	Unemployed	3 (15.00%)	1 (5.00%)	0.31
	Retired	15 (75.00%)	16 (80.00%)	0.71

Education	Completed high school	7 (35.00%)	9 (45.00%)	0.52
	Completed tertiary education	13 (65.00%)	11 (55.00%)	0.52

Affected eyes		27 (67.50%)	23 (57.50%)	0.36

Duration of OP (SD) (month)		5.17 (2.63)	5.24 (2.39)	0.93

Underlying disease	Diabetes mellitus	11 (55.00%)	13 (65.00%)	0.52
	Trauma	9 (45.00%)	7 (35.00%)	0.52

*Note*. OP: oculomotor paralysis.

**Table 2:** Comparison of response rate between two groups.

Groups	Response rate			Total (affected eyes)
	Complete response	Partial response	Nonresponse	
Acupuncture	4	16	7	27
Control	2	8	13	23

*Note*. Difference in efficacy between the two groups, *P* < 0.05.
